# Wounds of the Heart

**Published:** 1829

**Authors:** 


### II. Analytical.

30. *Wounds of the Heart.* In the number of this Journal for Oct. 1828, was published by Dr. Randall of Tennessee, the history of a case of gunshot wound of the lungs and heart. The patient lived 67 days from the accident and when examined after death the heart presented the following phenomena:

"It was, says Dr. R. considerably enlarged, and irregular on its surface, at the places where it adhered to the pericardium. In cutting though its parietes, we found some parts of them nearly cartilaginous, *and on opening the right ventricle, we were astonished to find three shot lying loose in its cavity.* This ventricle was greatly enlarged, and lined with a thick coat, from which there projected numerous papillæ of a dun colour, giving it the appearance of the upper surface of the tongue of an ox. *On opening the right auricle, we found two shot in its cavity, also lying detached.* The internal surface of the auricle did not appear to have sustained much injury from their presence. The shot had entered the heart about one third of the way from its base to its apex, the wounds made by them were at a little distance from each other; they had all cicatrized, but the spots were plainly to be seen.

In the last number of the American Journal of the Medical Sciences, (Aug. 1829,) Professor Coxe, one of its most learned contributors, has devoted an article to the facts quoted from Dr. Randall's paper, in which he admits the existence of the shot in the cavities of the heart, as reported by the author of the case, but expresses his opinion that they did not penetrate the heart when the boy was wounded, but made their way thither after death. The professor whose bibliothecal researches are, for America at least, profound and extensive, assures us, that the annals of medicine furnish abundant evidence that 'wounds of the heart are *invariably fatal* when penetrating its cavities;' and he cannot persuade himself that the case reported by Dr. R. constitutes an exception.

In support of this position he cites cases from a considerable number of writers, chiefly the older of the moderns, and expresses the belief that many others might be collected. As to the eschars found upon the external surface of the heart, the Professor believes them to have been produced by shot which did not penetrate its cavities. How then did those foreign bodies arrive at such an extraordinary goal? The following is his theory of this difficult phenomenon:—

May I ask, whether, in a wound, or rather five wounds penetrating the heart, little or no hæmorrhage would be likely to take place, either at the instant, or at some period before the adhesive process was completed; is it likely, in a wound made by the "whole charge" of shot, the greater part of which seem clustered in the lungs, that in the course of a week the wound would be healing, and in three or four weeks be completely cicatrized, while such perforations existed in the ever moving viscus? Is it likely that three shot would continue permanently during sixty-seven days in the right ventricle, and two in the auricle? and how indeed, from the location of the wounds, could the latter have gotten into the auricle? Are we to suppose, that during sixty-seven days, these five shot remained unmoved, exposed to the full torrent of the circulating stream? Would this rapid motion not rather have impelled them into the pulmonary artery; and driven them to a part whose calibre they could not pass; and when of course dissection might have traced them? In opposition to these difficulties, where is the dilemma, if we suppose, that, as the lungs were loaded in various parts with shot, the deranged state of those vascular parts, inducing suppuration, &c. should, (although the external wound might heal;) nevertheless, after that period, bring on hectic fever and other symptoms; and that some of the vessels, eroded or absorbed by the pressure of a leaden shot, might at length permit some of the cluster to fall into its cavity; and at the moment of death, by mere gravitation, find their way into the cavities of the heart, in which they were seen and detected? Had the idea suggested itself, it is not impossible, that even other shot might have been found, that might have fallen into the vena cava, and only been arrested by some distant valve!

Our chief object was to make known to the readers of this journal, the views of Professor Coxe, but we hope to be excused for recording the reflections which a perusal of them has excited.

1. Most of the cases which he has cited, were wounds by cutting instruments, and for an obvious physiological reason, these would much more certainly prove fatal than those made by shot.

2. No kind of wound would be so little likely to produce hæmorrhage from the cavities of the heart, as those made by shot when they were nearly spent, as must have been the case in this instance or they would have passed into the opposite side of the heart. The apertures which they would produce, in perforating a dense muscle, would be very small; and the extravasation which would instantly follow from the wounded vessels into the contused parts, would greatly contribute to close up the openings; which, moreover, on the known laws that govern the course of projectiles, would be more or less, sinuous.

3. The professor admits that shot had reached the outside of the heart and left eschars. But what became of these? It does not appear, that they were either buried in the substance of the heart, or fell back into the cavity of the pericardium.

4. If the shot penetrated the cavities of the organ, and the patient did not die of hæmorrhage, he would (if no other injury had been sustained) have lived, until their irritation on the new surfaces had raised a fatal inflammation.—Such an inflammation, if we may credit the report of the morbid appearances they did excite.

5. At first view we might suppose, that the cavities of the heart would have thrown the shot into the great vessels; but where is the evidence that they possess any such power?

The mode of contraction that would throw out the blood, might not eject small globular bodies of six times its specific gravity. On the contrary, it seems to us, that these bodies would most probably remain confined in their habitation, in perpetual contact with the inferior part of the parieties, from which, especially in the ventricle, it would certainly be difficult to dislodge them.

6. Professor Coxe asks how the shot could have gotten into the auricle? It certainly is not difficult to understand, that if they all penetrated the ventricle, they might have passed at the moment when it was receiving blood from the auricle, into that cavity, without wounding the ariculo-ventricular valves.

7. To the professor's explanation, there seem to us to be insuperable objections. If the shot came into the cavities of the heart after death, how can we account for the marks of inflammation which their surfaces exhibited? But what analogy have we for the erosion of arteries, in such manner as to admit the introduction of shot? Why did not aneurisms form when their parieties were partly ulcerated through, and why did not hæmorrhage take place at the moment when it was completed? The pulmonary artery and its ramifications receive and transmit blood to the last moment of existence, and if apertures were made into them large enough to admit of the introduction of shot by their own gravity, hæmorrhage *must* occur. Moreover, until the arteries had emptied themselves, the shot could not have passed along them to the heart; but they push forward their contents, after the heart has ceased to beat, by contracting upon them and it was not until after their elasticity had, finally, expanded them, to the size in which they appear in absolute death, that the shot could have begun to traverse them. But what, at that period could have moved those bodies to leave their resting places, *ascend* a branch of the pulmonary artery, and, after descending into the ventricle of the heart, mount upwards into its auricle? Finally, it appears from the account given, that neither abscess nor ulcer existed in the lungs.

Prosecuting the application of his theory, Prof. C. suggests that if a proper examination had been made, it might have been found, that other shot had fallen into the vena cava (ascendens) and only been arrested by some distant valve! But how an ulcer in the lungs or an absorption of the pulmonary tissue could have extended to that vessel, the Professor has not undertaken to instruct us; and till then we must suppose, that he refers to the *pulmonary* veins instead of the *vena cava.* He has even not informed us, whether the shot which made their way to the right chambers of the heart, took the route of the pulmonary artery or one of the venæ cavæ.

In conclusion, Prof. C. referring to his own explanation, remarks:—

By such, or some process of a similar description, can I alone suppose that we can *plausibly* explain the various circumstances of the case! The *actual* penetration of the cavities of the heart, is altogether problematical; and if the shot did really find their way in the manner affirmed by the gentlemen who relate the case; I can only say, that it is an anomaly as regards the wounds of the heart; and being in total opposition to *every* hitherto recorded fact, it must stand as an insulated one in the annals of medicine!

On this paragraph we would observe, that no just objection can be raised against the *facts* stated by Dr. R. from their being "insulated in the annals of medicine." It is an assumptoin to say, that all which may happen to the animal economy, has already happened; or that we can determine *a priori* on its conservative or recuperative resources. These are only to be known by observation and experiment, for the successful prosecution of which *incredulity* is not the only qualification required.

